# Case report: Inferior phrenic artery bleeding leading to retroperitoneal hematoma in a stillborn mother: diagnostic challenges and clinical implications

**DOI:** 10.1186/s12884-025-07772-0

**Published:** 2025-07-03

**Authors:** Xin Tian, Wenyao Li, Lianchang Liu, Duanrui Qiao

**Affiliations:** 1 The Second Hospital of Jilin University, No. 4026 Yatai Street, Nanguan District, Changchun City, Jilin Province China; 2 Jilin University, No. 2699 Qianjin Street, Changchun City, Jilin Province China

**Keywords:** Stillbirth, Interventional treatment, Retroperitoneal hematoma, Inferior phrenic artery, Case report

## Abstract

**Introduction:**

In this report, we present a unique case of a stillbirth accompanied by significant unexplained retroperitoneal hematoma (RPH).

**Patient Information:**

A 36-year-old patient at 31 6/7 weeks of gestation was confirmed stillbirth accompanied by clinical signs of significant blood loss.

**Diagnosis, Treatment and prognosis:**

After laparotomy and CTA examination, we clearly diagnosed the retroperitoneal hematoma. Arterial angiography revealed bleeding in the left inferior phrenic artery and an embolization was performed, successfully controlled the bleeding.

**Discussion:**

This case gives clinician a clue for searching for the causes in pregnant women presenting with unexplained bleeding and provides some tips for searching for the bleeding vessels of retroperitoneal hematoma. It also suggests that the huge retroperitoneal hematoma might be a cause of stillbirth.

## Introduction

Stillbirth, defined as the absence of life signs (no breathing, heartbeat, umbilical cord pulsation, or voluntary muscle movements) at birth in a fetus of ≥ 20 weeks gestation or a fetal weight of ≥ 350 g if the gestational age is unknown [1], is a profound event that impacts families and individuals. The etiology of stillbirth is often multifactorial, involving placental, maternal, fetal, and other unidentified factors. Common causes include placental insufficiency, maternal hypertension, infections, and fetal genetic abnormalities etc [2]. Despite these known factors, many cases remain unexplained, adding to the emotional and clinical complexity.

In this report, we present a unique case of a stillbirth accompanied by significant unexplained retroperitoneal hematoma (RPH). This case highlights the diagnostic challenges and the successful interventional treatment employed.

### Patient information

A 36-year-old patient at 31 6/7 weeks of gestation was admitted to our hospital with a diagnosis of stillbirth made two hours prior. One day before admission, she experienced severe, knife-like epigastric pain without an apparent cause. An obstetric ultrasound at a previous hospital showed no abnormalities. On the day of admission, decreased fetal movement was noted, and an obstetric ultrasound at the previous hospital confirmed the stillbirth. For further evaluation and management, she was referred to our hospital. The patient had no significant past medical history and had regular prenatal check-ups that showed no abnormalities.

Obstetric ultrasound: A single intrauterine fetus in cephalic presentation was observed. No fetal heartbeat was detected. Measurements: Biparietal diameter (BPD) 7.7 cm, head circumference (HC) 27.2 cm, abdominal circumference (AC) 27.0 cm, and femur length (FL) 5.8 cm. The placenta was attached to the anterior uterine wall with a thickness of 3.8 cm and classified as grade 0-I. Amniotic fluid: 17.4 cm. Ultrasound diagnostic impression: Intrauterine pregnancy, singleton, fetal demise, cephalic presentation, and nuchal cord.

Upon admission, the patient appeared anemic with pale conjunctiva, a heart rate of 140 bpm, and blood pressure of 118/68 mmHg. There were no visible signs of external bleeding such as vaginal, gastrointestinal, or urinary tract bleeding. Laboratory tests revealed a hemoglobin level of 76 g/L.

### Diagnosis, treatment and prognosis

The patient showed a sudden stillbirth and unexplained massive hemorrhage, leading to a poor general condition. An emergency cesarean section was planned by our obstetric team.

#### Surgical procedure

Under combined spinal-epidural anesthesia, the patient was placed in the supine position, and a urinary catheter was inserted. Standard surgical site disinfection was performed. A midline vertical incision, approximately 10 cm in length, was made in the lower abdomen. The skin and subcutaneous tissue were incised. A small incision was made in the anterior sheath of the rectus abdominis and extended bilaterally. The rectus abdominis muscle was separated, and a small incision was made in the peritoneum and extended bilaterally.

Upon entering the abdominal cavity, approximately 1000 mL of dark red, non-coagulated blood was observed. The lower uterine segment was well-formed. A transverse incision, approximately 10 cm in length, was made in the lower uterine segment, and the fetal membranes were ruptured, releasing a moderate amount of clear amniotic fluid, which was suctioned completely. The fetus was in the left occiput anterior (LOA) position and was manually delivered as a stillborn female. The umbilical cord was found to be wrapped around the neck, and was cut for further management.

Immediately after delivery, 20 units of oxytocin were injected into the uterine wall, and 1 mL of carbetocin was administered intramuscularly. The placenta and fetal membranes were delivered, and the uterine cavity was carefully cleaned. The incision was sutured layer by layer. Intraoperative exploration of the uterus and adnexa revealed no significant abnormalities. The abdominal cavity was thoroughly wiped and cleared, however, unexplained bleeding still exist. The surgical team extended the incision and performed a laparotomy, revealing a retroperitoneal hematoma approximately 15 cm × 10 cm. No active bleeding was detected from the liver, spleen, pancreas, kidney or gastrointestinal tract. A cardiovascular surgery consultation was requested intraoperatively, in order to rule out major vascular disease while identifying the bleeding site, the CTA was recommended.

#### CTA results and interventional procedure

**Fig. 1:**
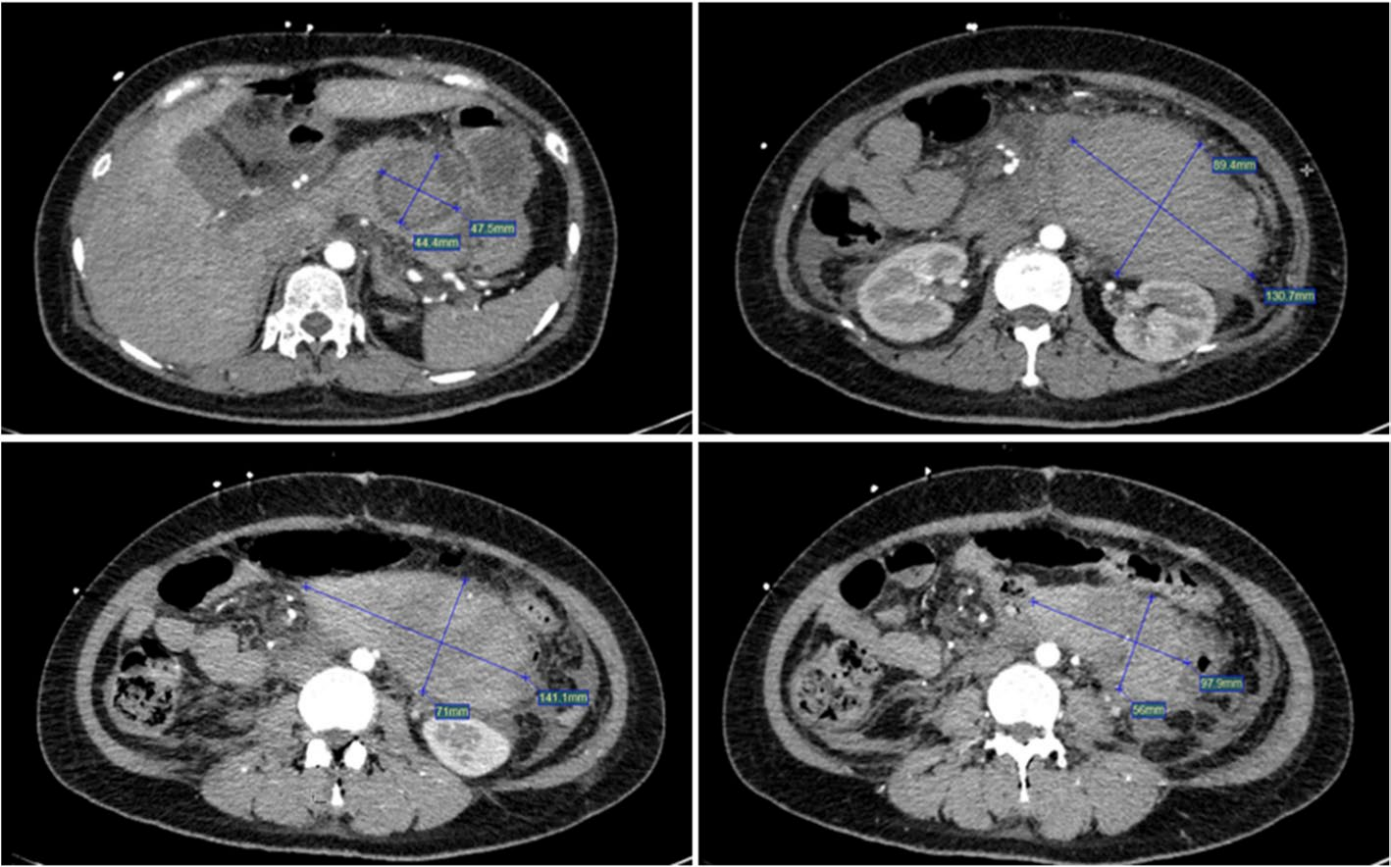
Preoperative CTA showed a huge RPH without a clear target vessel. Maximum cross-sectional area is approximately 13 cm × 8.9 cm. Average CT value: 67Hu

CTA revealed a large retroperitoneal hematoma of unclear origin (Fig. 1). Given to the patient's unstable vital signs, surgical intervention was deemed too risky. The interventional radiology team was consulted.

**Fig. 2:**
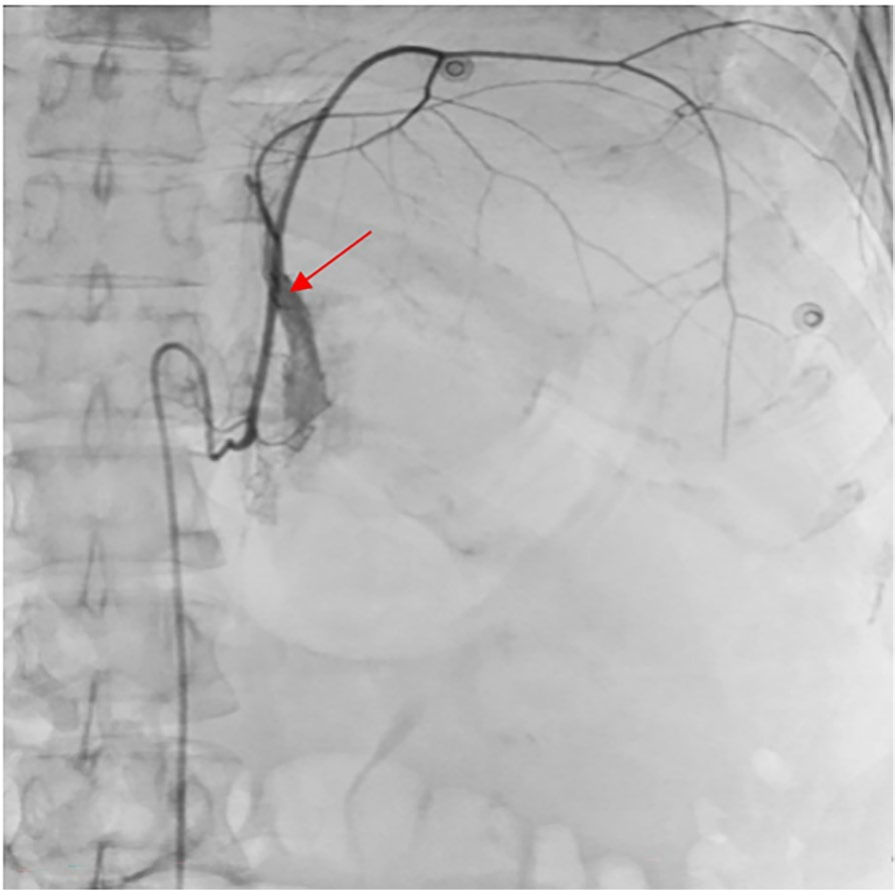
Angiography of left inferior phrenic artery showed multiple contrast medium extravasation

**Fig. 3:**
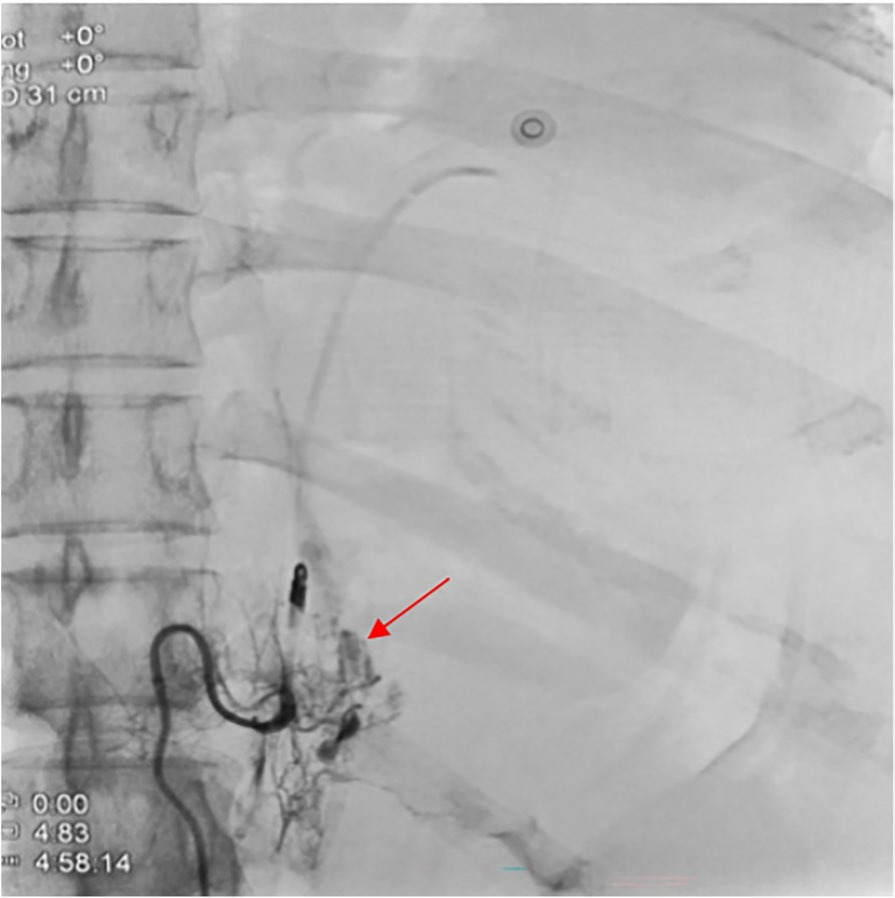
After embolization of the distal end by spring coils, the proximal end still showed a contrast medium extravasation

**Fig. 4:**
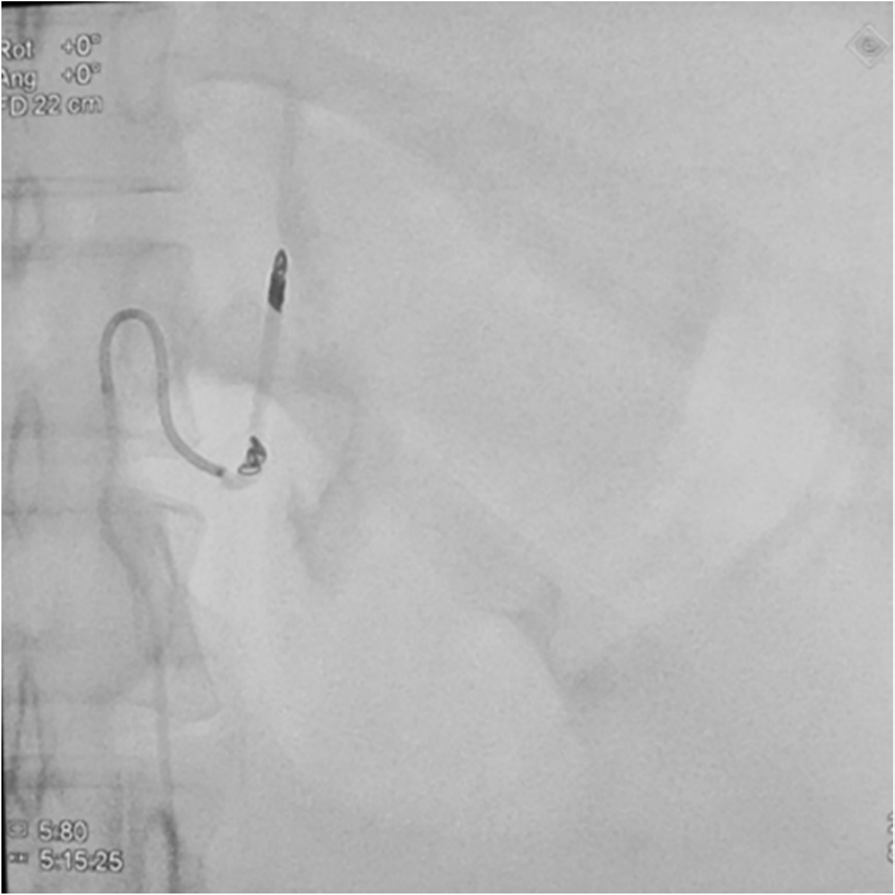
After embolization of the proximal end, the artery was occluded, and no contrast medium extravasation was revealed

We performed emergency intervention surgery for the patient. Using the Seldinger technique, the right femoral artery was punctured. Angiography of the hepatic, splenic, renal, left gastric, gastroduodenal, and superior mesenteric arteries showed no abnormalities. However, angiography of the left inferior phrenic artery revealed multiple sites of contrast medium extravasation. Embolization with gelatin sponge particles and micro-coils was performed, and post-embolization angiography showed no further contrast medium extravasation (Figs. 2, 3 and 4).

#### Prognosis

**Fig. 5:**
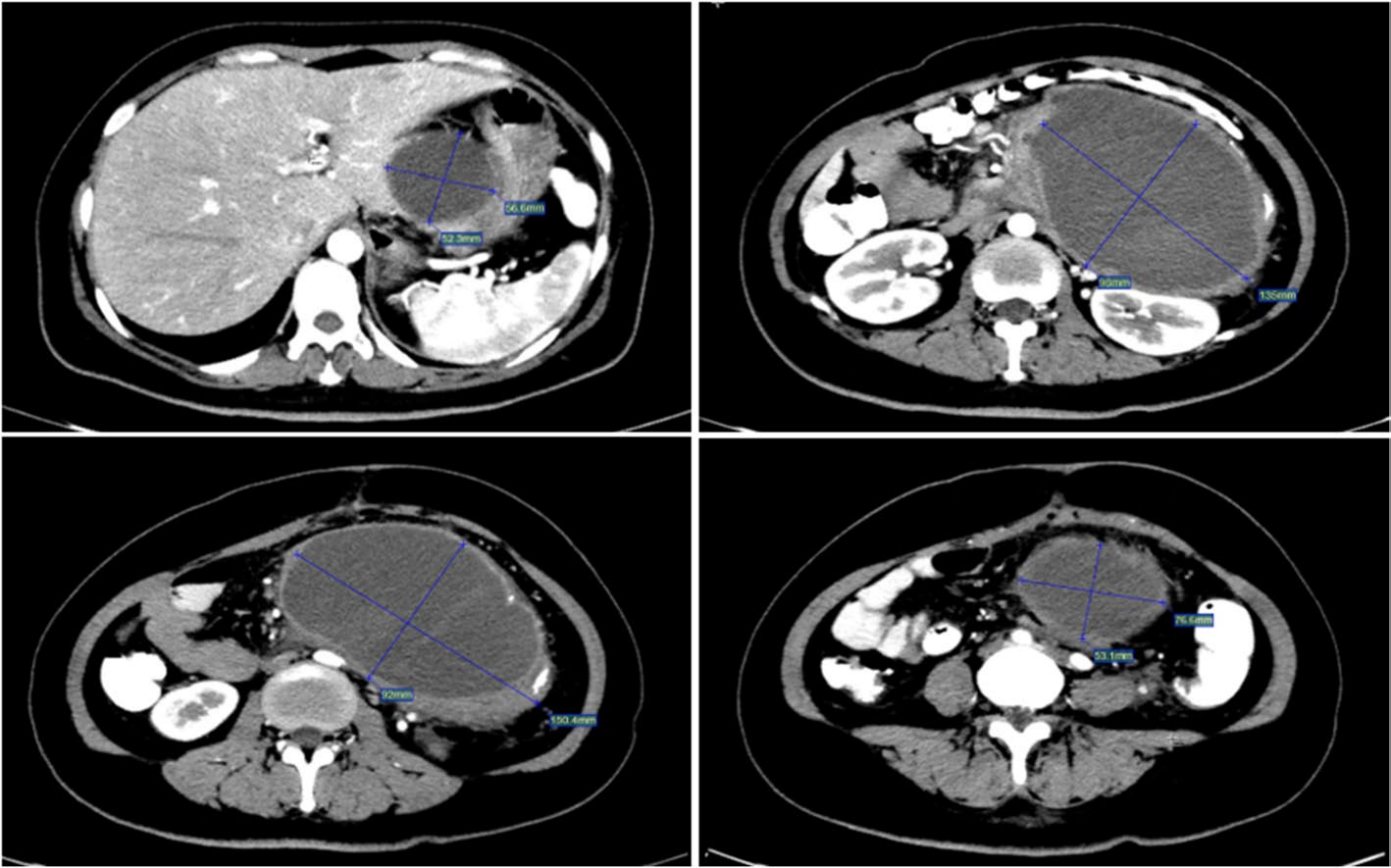
The density of the RPH showed a significant reduction. Maximum cross-sectional area is approximately 13.5 cm × 9.8 cm. Average CT value: 19Hu

Post-procedure, the patient's hemoglobin level improved to 102 g/L, and one week later it was 125 g/L. A follow-up abdominal CT scan 15 days post-procedure showed a significant reduction in hematoma density (Fig. 5).

## Discussion

Retroperitoneal hematoma (RPH) is a severe complication often secondary to pelvic fractures or injuries to retroperitoneal organs or vessels [3]. The retroperitoneal space is a potential cavity divided into three zones: I (central), II (peripheral), and III (pelvic) [4]. Clinical presentations of RPH include abdominal pain, palpable mass, skin bruising, hypotension, and hemoglobin drop, however, early symptoms may be nonspecific, leading to diagnostic challenges [5, 6].

Imaging, particularly CTA, is essential for diagnosing RPH and identifying bleeding sources [5]. Traditional treatment of RPH involved conservative measures such as fluid resuscitation and blood transfusions. However, a significant proportion of patients with RPH experience hemodynamic instability, in cases of rapid progression, surgical or interventional intervention may be necessary.

Interventional radiology has emerged as a crucial tool in managing RPH due to vascular injuries, offering a less invasive approach with lower surgical risk and high success rates. Interventional treatment efficacy is evaluated both technically (complete embolization of the target vessel) and clinically (improvement in patient condition) [7].

In this case, the patient's retroperitoneal hematoma was attributed to an injury of the inferior phrenic artery, a rare but serious cause of bleeding. Postoperatively, the patient recalled that a vomiting occurred after consuming something spicy the day before hospital admission. Some studies have reported that vomiting can lead to arterial injuries, including rupture of the left gastroepiploic artery, short gastric arteries, and renal artery, resulting in bleeding in corresponding area [8–10]. Vomiting may act as a triggering factor by exerting traction on associated ligaments, generating shear forces that ultimately lead to arterial rupture [10]. Therefore, we suppose the vomiting might have contributed to the rupture of the inferior phrenic artery.

This case gives the clinician a clue for searching for the bleeding vessels of retroperitoneal hematoma and emphasizes the importance of considering retroperitoneal hematoma in pregnant women presenting with unexplained bleeding and also demonstrates the effectiveness of interventional radiology in managing such complex cases. At the same time, this case also suggests that the huge retroperitoneal hematoma and resulting hemodynamic instability might be a cause of stillbirth.

## Data Availability

The data used and analysed during the current study are available from the corresponding author on reasonable request. All data generated or analysed during this study are included in this published article and its supplementary information files.
